# Chitosan nanoparticles-encapsulated cannabis extracts and their antimicrobial potential against skin pathogens

**DOI:** 10.3389/fmed.2025.1644502

**Published:** 2025-08-21

**Authors:** Tomáš Skala, Jordi Ventura, Ángela Morellá-Aucejo, Adéla Fraňková, Antoni Llopis-Lorente, Andrea Bernardos, Jan Tauchen, Zdeňka Kahánková, Vít Hubka, Pavel Klouček

**Affiliations:** ^1^Department of Food Science, Faculty of Agrobiology, Food and Natural Resources, Czech University of Life Sciences Prague, Prague, Czechia; ^2^Instituto Interuniversitario de Investigación de Reconocimiento Molecular y Desarrollo Tecnológico (IDM), Universitat Politècnica de València-Universitat de València, Valencia, Spain; ^3^Unidad Mixta UPV-CIPF de Investigación en Mecanismos de Enfermedades y Nanomedicina, Universitat Politècnica de València-Centro de Investigación Príncipe Felipe, Valencia, Spain; ^4^CIBER de Bioingeniería, Biomateriales y Nanomedicina (CIBER-BBN), Instituto de Salud Carlos III, Valencia, Spain; ^5^Department of Botany, Faculty of Science, Charles University, Prague, Czechia

**Keywords:** cannabis extracts, nanochitosan, encapsulation, antibacterial and antifungal, wound-healing

## Abstract

Cannabis compounds are well-known for their therapeutic applications in the treatment of various health issues. These substances, mainly cannabinoids, are known for their antimicrobial properties and ability to interact with various cells through endocannabinoid receptors. However, the limitations of cannabis extract, particularly its viscosity, stickiness, and low bioavailability when applied topically, limit its use in dermatology. To enhance topical applications for treating bacterial infections and dermatophytosis, cannabis extracts were encapsulated in chitosan nanoparticles, an easily accessible and cost-effective. Cannabis extracts were prepared from three cannabis strains differing in content of major cannabinoids, namely Chocolope (THCA-A), Jonas 1 (CBDA), and Hemp G (CBGA), and subsequently were encapsulated in chitosan nanoparticles. The resulting particles were characterized, and antimicrobial and cytotoxic activity was evaluated. The mean size of particles ranged from 89.1 ± 24.8 nm for empty nanoparticles to 355.6 ± 101.6 nm for particles containing Hemp G extract. Considering the extract:chitosan ratio (1:10 w/w, 1:20 w/w respectively) and the encapsulation efficiency (EE) range from 44.65 ± 4.39% to 94.44 ± 0.93%, total amount of extracts encapsulated in chitosan nanoparticles ranged from 2.96 ± 0.05 to 5.61 ± 0.19% in 1 g of chitosan nanopowder. Most significant antimicrobial effect was observed against the fungi *Nannizzia fulva* CCF 6025, where the MIC_80_ of the pure extract from Jonas 1 variety was 256 μg/mL while the encapsulated extract in chitosan nanoparticles (1:10 w/w extract:chitosan ratio) inhibited growth at a concentration of 256 μg/mL of nanoparticles (corresponding to 13.05 ± 0.13 μg/mL of extract). Overall, encapsulation reduced the amount of extract required to inhibit the growth of pathogenic microorganisms by up to several times, notably in case of dermatophytes, compared to non-encapsulated extracts. Encapsulation also reduced the cytotoxic effects of the extracts on human keratinocytes. Furthermore, pure high-THCA-A extract and encapsulated extract in chitosan nanoparticles slightly increased cell viability after 72 h exposure in low concentrations compared to control. These results may suggest the chitosan nanoparticles-encapsulated formulations as a suitable topical delivery form of cannabis extracts, offering a possible adjunctive treatment of dermatophytosis and wound healing.

## Graphical abstract

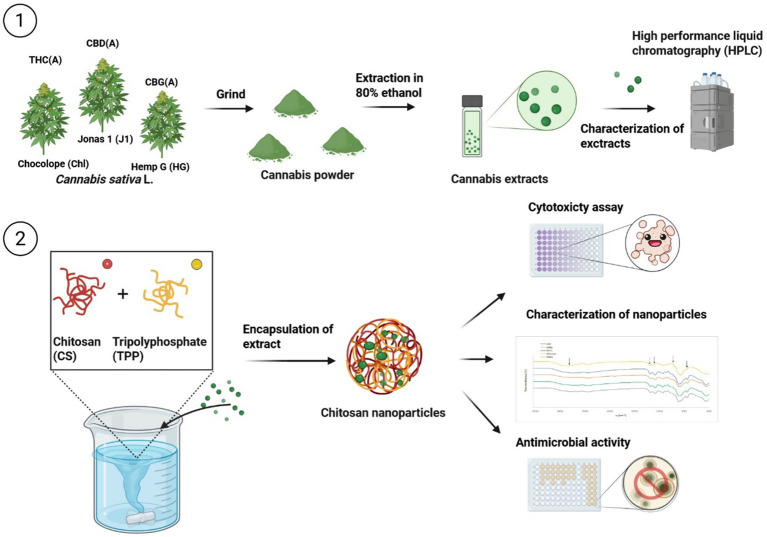

## 1 Introduction

Skin is one of the largest human organs in terms of size and surface (1). It is inhabited by various microorganisms such as viruses, bacteria and fungi that live in homeostasis (2). However, the disruption of the skin microbiome can lead to the development of multifarious diseases which are, at the global level, rated as the fourth leading cause of non-fatal disease burden including fungal skin diseases, acne, impetigo, etc. (3).

The most frequent causative agents of skin diseases are bacteria and fungi. Bacterial infections are commonly caused by opportunistic pathogens, e.g., *Staphylococcus aureus*, *Staphylococcus epidermidis*, and *Streptococcus pyogenes* that can cause painful skin infections such as impetigo, folliculitis, etc. Rarely, they can cause much more serious illnesses, such as endocarditis, streptococcal toxic shock syndrome, sepsis or staphylococcal scalded skin syndrome, eventually leading to death (4, 5).

Superficial fungal diseases (dermatomycosis) are predominantly caused by fungi from the genus *Trichophyton* spp., *Microsporum* spp., *Nannizzia* spp. and *Epidermophyton* spp. As a result of increasing globalization and associated human migration, it is estimated that 20–25% (6, 7) of the world‘s population suffers from some type of dermatomycosis and this number is gradually increasing (8). For example, in some parts of India, nearly 60% of the population is suffering from certain type of skin disease (9). The symptoms of fungal infections are usually painless but may be accompanied by unpleasant itching and redness of the affected area. On the other hand, fungal skin diseases can significantly negatively impact the psychological and social well-being of the patient (10).

Most skin diseases are treated with topical or systemic antibiotics, or their combination in case of serious infections (11). Unfortunately, the widespread use of synthetic antibiotics significantly increases the resistance of bacteria and fungi to these drugs, mainly due to overuse in intensive livestock farming (12). New strains of bacteria resistant not only to conventional antibiotics but also to last-resort antibiotics, such as methicillin-resistant *S. aureus* (MRSA) (13) are being discovered every year (14). The situation is no less alarming in the case of dermatophytes. In 2020, for example, 71% of terbinafine-resistant *Trichophyton* spp. clinical isolates were identified in India (15) and these resistant dermatophytes are spreading worldwide (16). The resistance of microorganisms to commonly used antibiotics is expected to increase dramatically in the coming years. The high cost of their production and the wide range of side effects must also be considered. The above information gives great impetus to the search for new remedies that are less toxic and more accessible. A solution could be offered by certain plants and their products.

Plants have been used to treat diseases since time immemorial. Dried cannabis flowers were used in ancient times for a number of diseases, including skin infections (17). The results of current research have demonstrated the effects of certain substances from cannabis being effective not only against diseases such as lupus or psoriasis (18, 19) but also against microbial pathogens causing skin infections such as acne (20). However, current knowledge of the whole plant extracts activity is limited.

Cannabis contains many bioactive compounds across the chemical spectrum, but the predominant antimicrobial effect is attributed to the cannabinoids, particularly cannabidiol (CBD) (20), cannabidiol acid (CBDA) (21) cannabigerol (CBG) (22), cannabigerol acid (CBGA) (23), Δ9-tetrahydrocannabiol (THC) (24). Antimicrobial activity has also been reported for THCA (tetrahydrocannabiol acid) and cannabinol (CBN) (21, 25). Furthermore, antimicrobial effects are also attributed to terpenes such as *β*-caryophylene, limonene, *α*-pinene, β-pinene, or myrcene, commonly abundant in cannabis plants (26, 27). On top of that, a possible synergistic effect has been described between cannabinoids and terpenes, which may contribute to higher therapeutic efficacy of extracts (28).

In plants, cannabinoids are mostly present in the form of non-psychoactive acid precursors (CBDA, CBGA, THCA), which are converted to a neutral form through thermal processes called decarboxylation (29). The composition and effects of cannabis extracts depend on the extraction method used but above all on the chemotype of the cannabis plant (28). Cannabis is commonly prescribed for topical application in the form of creams, tinctures or extracts. However, the extract is usually not thermally processed, hence most cannabinoids are present in their acid form. In addition, increasing trend among patients to use cannabis products without previous heating is currently observed (30), mainly due to the better dosing (31). However, difficulties can be encountered in their topical application due to their highly rigid and sticky consistency with low transdermal bioavailability (32). Here, we propose encapsulation into a nano-based carrier as a possible solution to improve handling and increase the bioavailability of present cannabinoids. In particular, we focus on the use of chitosan nanoparticles.

Chitosan (CS) is a natural nontoxic and biodegradable copolymer of *β*(1,4)2-amino-2-deoxy D-glucose and *N*-acetyl D-glucosamine in variable ratios, derived by deacetylation of chitin through chemical enzymatic processes (33). It is readily available from renewable sources and offers valuable properties such as antibacterial and antifungal activity, depending on the degree of deacetylation. In addition, the application of chitosan to the skin has other benefits, such as support to wound healing, hydrating properties, and water loss prevention. Nano-sized chitosan has the same properties as normal chitosan but offers a larger active surface area and enhanced antimicrobial activity. Additionally, it can boost the antimicrobial activity of the encapsulated compounds, as demonstrated for example with lupulone and xanthohumol from hops (34) or *Mentha longifolia* leaf extract (35). Furthermore, it may enhance their bioavailability by improving targeting and solubility (36).

This study aimed to prepare and characterize chitosan nanoparticles (NP) containing extracts from three cannabis chemovars, evaluate and compare the antimicrobial activity of pure and encapsulated extracts against bacteria and fungi, and determine the cytotoxic effects of (non)encapsulated extracts on human skin cells.

## 2 Materials and methods

### 2.1 Chemicals

All solvents used for the GC and HPLC analysis were of analytical grade. Acetonitrile (ACN) and formic acid (FA) together with terpene standards [(+)-3-carene, (+)-limonene, *α*-bergamotene, α-pinene, α-terpineol, ß-myrcene, camphene, *β*-caryophyllene, caryophyllene oxide, farnesene mix, fenchol, humulene, linalool, terpinolene] and *n*-alkane (C_8_-C_30_) standard solutions were purchased from Sigma-Aldrich (Prague, Czechia). Methanol (MeOH), *n*-hexane, dimethyl sulfoxide (DMSO), sodium hydroxide and ethanol (EtOH) were obtained from VWR Chemicals (Prague, Czechia). Standards of cannabinoids, namely, cannabidivarin (CBDV), cannabidivarinic acid (CBDVA), cannabigerol (CBG), cannabigerolic acid (CBGA), cannabinol (CBN), cannabinolic acid (CBNA), cannabidiol (CBD), cannabidiolic acid (CBDA), cannabichromene (CBC), tetrahydrocannabivarin (THCV), 19-tetrahydrocannabinol (THC), and tetrahydrocannabinolic acid A (THCA-A) were purchased from Cayman Chemicals (Ann Arbor, United States). Chitosan (practical grade), sodium tripolyphosphate (TPP) and acetic acid were purchased from Sigma-Aldrich. Microbiological growth media Mueller-Hinton Broth (MHB), Sabouraud dextrose agar (SDA) and Sabouraud dextrose broth (SDB) were bought from OXOID (Prague, Czechia), antibiotics clotrimazole (CLT), chloramphenicol (CLP), ampicillin (AMP), terbinafine (TB) were obtained from Sigma-Aldrich. Human spontaneously immortalized keratinocyte cell line (HaCaT) was obtained from Cell Lines Service GmbH (Eppelheim, Germany), Dublecco’s modified eagle medium (DMEM), Dublecco’s phosphate buffered saline (DPBS), penicillin:streptomycin and enzyme accutase were purchased from Biowest (Nuaillé, France). Fetal bovine serum (FBS) was bought from Biosera (Prague, Czechia), and 3-(4, 5-dimethylthiazol-2-yl)-2, 5-diphenyl-2H-tetrazolium bromid (MTT) was obtained from Sigma-Aldrich.

### 2.2 Preparation and characterization of cannabis extracts

Cannabis ethanolic extracts were prepared from three cannabis strains differing in cannabinoid profile. Namely Chocolope (Chl) (DNA Genetics, NL) with high THC(A) content, cultivated under controlled indoor conditions at the Department of Food Science, Faculty of Agrobiology Food and Natural Resources, Czech University of Life Sciences Prague in 2020. High CBD(A) strain with the working name “Jonas 1, J1” was obtained from Phyto Hemp s.r.o. (Czech Republic) as well as cultivar with high CBG(A) content (working name “Hemp G, HG”). These two genotypes were grown outdoor.

The extracts were prepared by maceration of 60 g of dried homogenized cannabis inflorescences for 48 h in 80% ethanol in the ratio 6:1 (solvent: flower; v/w). Subsequently, the extract was filtered, and the solvent evaporated using a Rotavapor® R-100 vacuum evaporator (Buchi, CHE) at 40 °C. Prepared extracts were stored at −20 °C. The cannabinoid profiles of prepared extracts were determined by HPLC/DAD (Thermo Fisher Scientific, USA). The terpene profile was determined by GC/MS (Agilent, USA). Both methods were described in detail in a previous study (37).

### 2.3 Preparation of empty and cannabis extract-loaded chitosan nanoparticles

Chitosan nanoparticles were prepared by the ionotropic gelation (38) with slight modifications. Chitosan was dissolved at 0.5% w/v in 1% acetic acid (v/v). After 10 min of ultrasonication, the solution was stirred for 3 h until complete dissolution. Subsequently, pH was raised to 5.0 by 10 N sodium hydroxide and the solution was stirred for an additional 30 min. In the next step 0.25% TPP in distilled water was added to reach the required mass ratio of CS: TPP 3:1 (w/w), and the solution was stirred for another 30 min. Chitosan nanoparticles were formed immediately after the addition of TPP. The solution containing nanoparticles was centrifuged at 9000 g for 45 min at 4 °C. The obtained pellets were extensively rinsed with distilled water to remove all the residues of sodium hydroxide. Finally, the pellets were freeze-dried (Gregor Instruments, Czech Republic), weighed, ground and stored at 4 °C for further use.

To prepare chitosan nanoparticles with three different cannabis extracts, 2 mL of cannabis extracts (c = 50 mg/mL) in 80% ethanol were added to 200 or 400 mL of CS solution with 1% of acetic acid after 10 min of ultrasonication and adjusted pH to 5.0. The extract: CS ratios were selected based on physicochemical properties of crude extracts and it corresponds to 1 g and 2 g of CS, or 1:10 and 1:20 w/w extract: CS ratio, respectively. After that the same procedure as described above was followed. The supernatants from centrifugation were kept for subsequent indirect determination of the encapsulation efficacy. In total, seven types of nanoparticles were prepared (Table 1).

**Table 1:** The name and extract: CS ratio of the prepared nanoparticle batches.

Working name	Extract	Extract: chitosan ratio (w/w)	Amount of extract (mg)	Amount of chitosan (g)	Volume of solution (mL)
CNP	–	–	–	2	400
CNT1	Chocolope	1:10	100	1	200
CNT2		1:20	100	2	400
CNB1	Jonas 1	1:10	100	1	200
CNB2		1:20	100	2	400
CNG1	Hemp G	1:10	100	1	200
CNG2		1:20	100	2	400

Dissolved TPPin distilled water at a concentration of 0.25% was added to a solution of CS containing 1% acetic acid to achieve a final CS: TPP ratio of 3:1 (w/w). CNP, empty chitosan nanoparticles; CNT1 and CNT2, Chocolope chitosan nanoparticles; CNB1 and CNB2, Jonas 1 chitosan nanoparticles; CNG1 and CNG2, Hemp G chitosan nanoparticles.

### 2.4 Characterization of the nanoparticles

Particle size distribution, zeta potential and polydispersion index (PDI) of prepared empty and loaded nanoparticles were measured by DLS (Dynamic Light Scattering) with a Zetasizer Nano SZ instrument (Malvern Instruments, UK). The analysis was performed at a scattering angle of 173°, at a temperature of 25 °C, using 2 g/L solution of reconstituted nanoparticles (previously dried and ground to a fine powder) in deionized distilled water, at pH 5. The morphology of the nanoparticles was observed using a field emission scanning electron microscope (FE-SEM) Zeiss Ultra 55 (Zeiss, Germany) at 5 kV and 45 mA. Before observation, the samples were coated with platinum. Fourier transform infrared spectroscopy (FTIR) spectra were taken on Bruker Tensor 27 spectrometer (Bruker, UK). Finely, ground samples were placed on the ATR crystal, and 32 consecutive scans were performed on each sample. Spectra were taken from 4,000 to 400 cm^−1^ and the resolution of the wavenumber was 2 cm^−1^. Encapsulation efficiency (%EE) was determined indirectly from the supernatant obtained during the centrifugation. Supernatant was filtered through a 0.1 μm syringe filter (Milipore, USA), evaporated under the stream of nitrogen, diluted in MeOH, analyzed by HPLC/DAD and the EE was calculated according to Equation 1:


(1) $$\%\mathrm{EE}=\frac{\begin{array}{c} \text{total added cannabinoid content}\\ -\text{cannabinoid content in supernatant}\;\; \end{array}}{\text{total added cannabinoid content}}x100$$

### 2.5 Determination of the antibacterial and antifungal activity of (un)loaded nanoparticles

*In vitro* antimicrobial and antifungal activity was determined by broth microdilution methods according to the Clinical and Laboratory Standards Institute (CLSI) M07-A8 and M38-A2 for bacteria and fungi, respectively (39, 40), with slight modifications. The results were in both cases expressed as minimum inhibitory concentration (MIC_80_), i.e., the lowest concentration that inhibited bacterial or fungal growth by 80% compared to the untreated control. All experiments were carried out in 3 technical and 3 independent replicates. Antimicrobial activity of the empty and loaded nanoparticles was tested against 7 pathogenic bacterial strains, namely *Staphylococcus aureus* ATCC 25923 and 29213, *S. epidermidis* CCM 50 and 4418, *S. lugdunensis* CCM 4069, *S. saprophyticus* CCM 2727 and *Streptococcus pyogenes* CCM 4425, While their antifungal activity was determined against 12 dermatophytes, namely *Arthroderma insingulare* (CCF 5417; 5943), *Epidermophyton floccosum* CCM 8339, *Microsporum gypseum* CCM 8342, 3 strains of *Nannizzia fulva* (CCF 6025; 5338; 5782), *Nannizzia gypsea* CCF 5215, two strains of *Trichophyton rubrum* (CCF 4934; 4879), *Trichophyton interdigitale* CCM 8377 and *Trichophyton tonsurans* CCF 4930. The strains were purchased from the American Type Culture Collection (ATCC), Czech Collection of Microorganisms (CCM), or kindly provided by the Culture Collection of Fungi, Department of Botany, Charles University, Prague (CCF).

#### 2.5.1 Determination of minimal inhibitory concentration for bacteria

Each batch of nanoparticles was resuspended in MHB to a final concentration of 1,024 μg/mL. The suspension was mixed with an Ultra turrax (IKA T25, Germany) at 15.000 rpm for 15 s to create a homogenous solution. Subsequently, the two-fold serial dilution of the loaded NP was prepared at concentrations ranging from 8 to 1,024 μg/mL to 96 microtiter plates containing MHB as a growth medium. Standardized inocula with a final density of 0.5 McF (1–2 × 10^8^ CFU/mL) prepared from 1-day-old bacterial cultures cultivated in MHB at 37 °C were used for microtiter plates inoculation. The MIC_80_ was determined after 24 h cultivation at 37 °C using the BioTek Synergy H1 reader (Agilent, US) at 512 nm. Both negative (broth and empty nanoparticles) and positive (chloramphenicol and ampicillin) controls were also prepared.

#### 2.5.2 Determination of minimal inhibitory concentration for dermatophytes

The nanoparticles were resuspended in SDB to a final concentration of 1,024 μg/mL. Subsequently, suspensions were mixed with Ultraturax at 15.000 rpm for 15 s and two-fold serial dilution at concentrations ranging from 8 to 1,024 μg/mL to 96 microtiter plates containing SDB as growth medium was prepared. The microtiter plates were inoculated with fresh dermatophyte inocula prepared from 8–12 days old growing cultures on SDA at 27 °C at density 4–5 × 10^5^ CFU/mL. After 5 days of cultivation at 27 °C, the MIC_80_ was determined by microplate reader BioTek Synergy H1 at 512 nm. Broth, empty nanoparticles and antibiotics (clotrimazole, terbinafine) were used as negative and positive control, respectively.

### 2.6 Evaluation of the cytotoxic effects of chitosan nanoparticles on human keratinocytes using the MTT assay

Human keratinocytes cell line HaCaT (CLS, Germany) was maintained at 37 °C in a controlled atmosphere with 5% CO_2_ and 95% humidity. Cells were cultivated in DMEM medium supplemented with penicillin (100 U/mL) and streptomycin (100 μg/mL) in T75 cm^2^ flasks with surface treatment. HaCaT cells were sub-cultured by detachment using enzyme accutase at 80–90% confluency every 3rd or 4th day and fresh medium was replenished every 2–3 days.

The cytotoxic effect of crude and seven encapsulated extracts was determined by the MTT cell viability assay (41). Cells in the exponential growth phase were detached at 80–90% confluency and viable cells were counted using trypan blue solution in a Neubauer counting chamber under the microscope (Motic AE 2000, Spain). Cells were seeded in 96-well surface treated plates at 1.25 × 10^5^ cells/mL in concentration 200 μL/well and left in an incubator for 24 h. After that, the cells were treated with serially diluted particles/extracts/ATB in concentrations ranging from 4 to 1,024 μg/mL and incubated for 24, 48 and 72 h. After the required incubation period, the medium in all experimental groups, including controls, was aspirated and cells were washed twice with 200 μL of DPBS. Subsequently, 200 μL of serum free DMEM containing 500 μg/mL of MTT was added to the experimental and control wells. The plates were incubated for an additional 2 h in the dark. Subsequently, medium was aspirated and formed formazan crystals were dissolved in 200 μL of DMSO, and the absorbance was then measured using BioTek Synergy H1 microplate reader at 540 nm. The results were expressed as a 50% inhibition of viability (IC_50_) compared to the untreated control. Untreated cells were used as a control group and pure DMSO as blank control. All experimental groups and concentrations were tested in pentaplicates, whereas control group was tested in eight replicates. The degree of cytotoxicity was evaluated according following criteria: IC_50_ ≤ 20 μg/mL = high cytotoxic effect, IC_50_ between 21 and 200 μg/mL = moderately cytotoxic, IC_50_ ranged from 201 to 500 μg/mL = weakly cytotoxic and IC_50_ ≥ 501 μg/mL = no cytotoxicity (42).

### 2.7 Statistical analysis

The results were expressed as mean values and standard deviation (SD) in Excel and STATISTICA 12 software (StatSoft, Tulsa, United States). One-way analysis of variance (ANOVA) followed by Tukey’s HSD Test with *p* = 0.05 was performed.

## 3 Results and discussion

Prepared ethanol extracts from three cannabis varieties differing in cannabinoid and terpene content demonstrated antimicrobial activity against all tested pathogens ranging from 4 to 512 μg/mL and relatively high cytotoxicity against human keratinocytes. The extracts were subsequently incorporated into chitosan and the resulting nanoparticles were physically and chemically characterized and subsequently retested for their antimicrobial and cytotoxic activity. Chitosan nanoparticles containing Chocolope extract (1:10 extract:chitosan w/w ratio) exhibited the highest antimicrobial activity, whereas particles containing Hemp G extract showed the poorest activity.

### 3.1 Chemical characterization of cannabis extracts

The total content of cannabinoids ranged from 174.43 mg/g for HG to 346.04 mg/g for Chl, respectively (Table 2). The cannabinoid profile varied according to the strain, THCA dominated in Chl, CBDA in J1 and CBGA in the Hemp G strain. In the case of J1, CBGA was also present in significant amounts. The remaining cannabinoids were present only in minor or trace amounts. This corresponds to the natural dominance of major cannabinoids described above (28). The terpene profile of the extracts was very poor, except for Chl strain. *β*- and *γ*- eudesmol were the most dominant ones in Hemp G and Jonas 1 strains. The major terpens in Chl strain were β-caryophyllene and β-eudesmol (Table 3).

**Table 2:** Cannabinoids content in extracts (mg/g).

**Cannabinoids**	**Cannabis strain**		
	**Chocolope**	**Jonas 1**	**Hemp G**
CBC	1.33 ± 0.14	1.36 ± 0.02	0.99 ± 0.05
CBD	0.13 ± 0.01	23.98 ± 2.14	0.72 ± 0.02
CBDA	4.68 ± 0.10	231.32 ± 17.69	9.87 ± 0.33
CBDV	0.18 ± 0.01	0.14 ± 0.01	0.19 ± 0.02
CBDVA	1.32 ± 0.12	0.85 ± 0.11	0.78 ± 0.05
CBG	1.73 ± 0.07	4.44 ± 0.03	7.61 ± 0.53
CBGA	10.59 ± 0.32	52.19 ± 4.72	152.21 ± 7.06
CBN	0.42 ±0.02	0.08 ± 0.01	0.05 ± 0.01
THC	51.72 ± 4.55	1.44 ± 0.19	0.31 ± 0.02
THCA-A	273.43 ± 23.54	7.72 ± 0.23	1.24 ± 0.09
THCV	0.50 ± 0.02	0.01 ± 0.00	0.45 ± 0.03
TOTAL CANNABINOIDS	346.04 ± 28.90	323.55 ± 25.15	174.43 ± 8.20
Extract yield (%)	11.15	9.57	6.98

Results are presented as mean ± standard deviation. CBC, cannabichromene; CBD, cannabidiol; CBDA, cannabidiolic acid; CBDV, cannabidivarine; CBDVA, cannabidivarinic acid; CBG, cannabigerol; CBGA, cannabigerolic acid; CBN, cannabinol; THC, Δ^9^-tetrahydrocannabinol; THCA-A, tetrahydrocannabinolic acid; THCV, tetrahydrocannabivarin.

**Table 3:** Relative ratio (%) of identified terpenes in cannabis strains.

				**Cannabis strain relative ratio (%) of identifies compounds**		
**Compound**	**rt (min)**	**RI**	**RI lit**	**Chocolope**	**Jonas 1**	**Hemp G**
limonene	12.1	1036	1030	0.79		
2-octenal	13.1	1074	1062	0.27		
linalool	13.9	1103	1101	7.04		
fenchol	14.5	1125	1124	2.30		
trans-2-pinanol	14.8	1134	1132	1.64		
endo-borneol	16.0	1179	1179	0.68		
α-terpineol	16.6	1201	1191	2.81		
α-bergamotene	22.6	1447	1438	5.39		
caryophyllene	22.9	1441	1423	18.58	6.25	
cis-β-Farnesene	23.4	1462	1459	7.20	0.57	
humulene	23.8	1476	1477	6.62	1.96	
epi-β-selinene	24.6	1509	1509	2.75		
β-bisabolene	24.8	1518	1511	2.65	1.49	
sesquicineole	25.0	1527	1521		0.20	
X1	25.2	1544		1.20	0.11	
X2	25.7	1557			2.22	
selina-3,7(11)-diene	25.8	1563	1545	9.50	1.49	
X3	26.3	1582		1.07		
X4	26.9	1610				7.22
guaiol	27.1	1617	1616	7.64	5.73	12.66
γ - eudesmol	27.7	1644	1630		45.17	
ß-eudesmol	28.4	1674		14.43	21.94	55.82
X5	28.7	1689		3.67		
α-bisabolol	28.9	1694	1685		7.57	
X6	29.2	1707		0.66		
X7	29.5	1722		0.09		
X8	30.4	1766		0.61		
X9	30.8	1785		0.40		
X10	31.1	1797			1.73	
cis-eudesm-6-en-11-ol	31.6	1822	1821	1.39	2.08	12.19
selinane-4α,11-diol	31.8	1836	1822		1.48	12.13

rt, retention times; RI, calculated retention index; RI lit, retention index given in the literature; X1–10, not identified terpenes. The results are expressed as relative ratio of area compound to total area of identified compounds.

### 3.2 Characterization of nanoparticles

**Figure 1:**
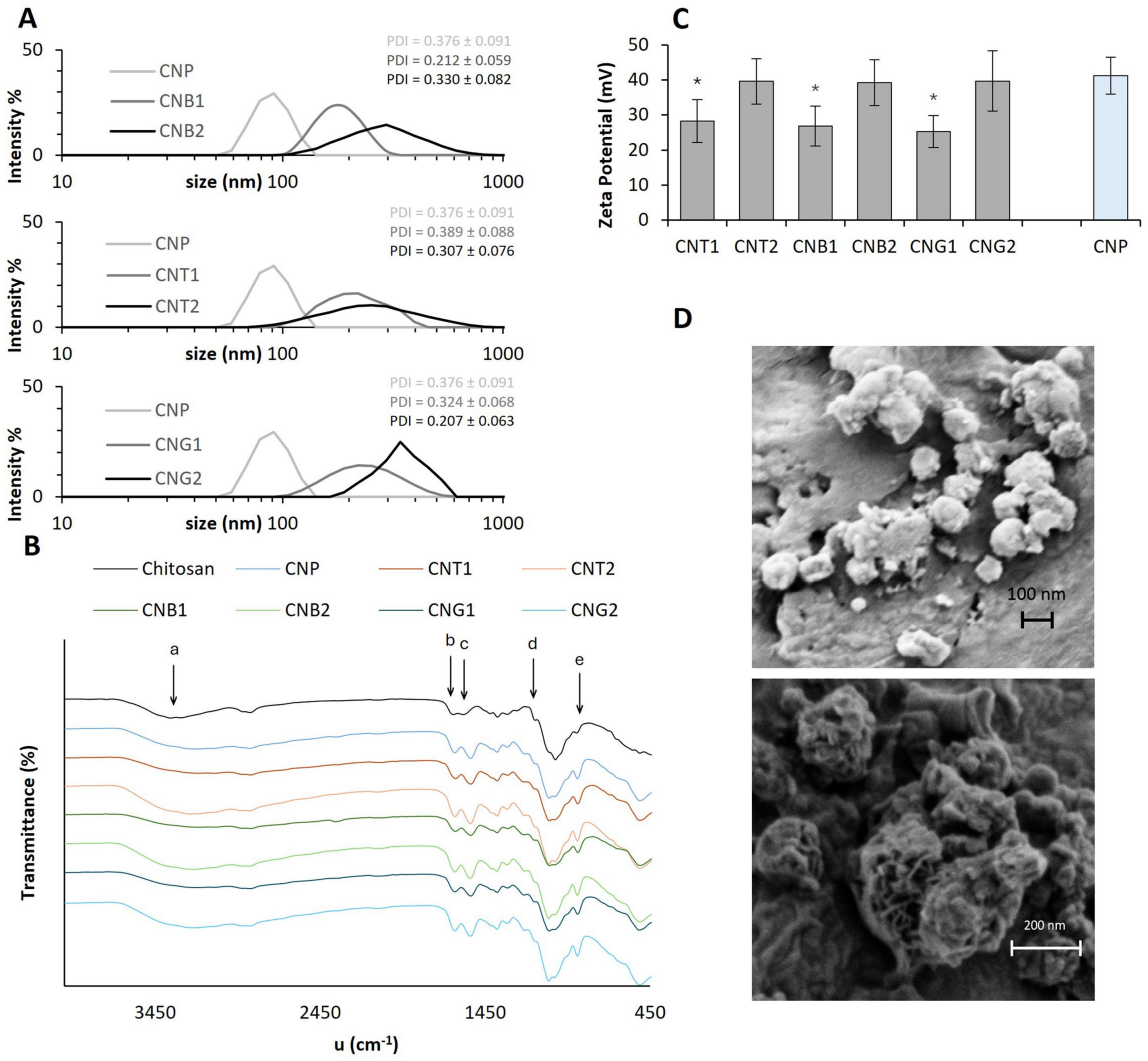
Hydrodynamic diameter (nm) with PDI **(A)**, FT-IR spectra **(B)**, Zeta potential (mV) with its errors – values significantly different from the CNP are marked with asterisks (*p* < 0.05) **(C)**, FE-SEM image. Upper image: CNP (Mag = 22.86 K X, ESB Grid = 0 V, WD = 5.5 mm); Bottom image: CNT1 (Mag = 75.00 K X, ESB Grid = 0 V, WD = 3.4 mm) **(D)**. CNP, empty chitosan nanoparticles; CNT1 and CNT2, Chocolope chitosan nanoparticles; CNB1 and CNB2, Jonas 1 chitosan nanoparticles; CNG1 and CNG2, Hemp G chitosan nanoparticles.

The prepared particles had a form of a white to green fine powder with a mild odor of cannabis. The mean size of the chitosan nanoparticles with a slightly broad size distribution ranged from 89.1 nm for empty particles to 355.6 nm for CNG2 particles (Figure 1A). The size of particles increased with the addition of the extract. However, this effect was observed primarily in particles with a lower extract content, which may be attributed to the interaction between polymers and extract composition. These findings are consistent with a previous report (35). The range of PDI was below 0.4 (Figure 1A), indicating uniformity of the prepared particles (43). In some batches of nanoparticles, several formations of aggregates >400 nm have been observed, probably due to presence of impurities (dust) or due to the disruption of the equilibrium between the hydrogen bond attractions and the electrostatic repulsions between chitosan nanoparticles. These aggregates may exhibit distinct physicochemical properties that could affect the biological activity of the encapsulated extracts. However, the formation of these large microparticles can be restricted by increasing the CS: TPP ratio, or a higher volume of the reaction medium, i.e., water (44).

The infrared analysis of CS, and NPs was performed to characterize the chemical structure of nanoparticles (Figure 1B). FTIR spectra of CS exhibited two vibrations at 1655 cm^−1^ and 1,573 cm^−1^, which were attributed to the CONH_2_ and NH_2_ groups, respectively. The intensity of those functional groups decreases, and two new peaks appear at 1637 cm^−1^ (b) and 1,543 cm^−1^ (c) after the addition of TPP. That proves the crosslinking of ammonium groups with TPP, which corresponds with similar results in a study by Lifeng et al. (38). The broader region starting at 3292 cm^−1^ (a) in CNP and loaded NPs compared to CS could be correlated with enhanced hydrogen bonding. Furthermore, the appearance of a peak at 1250 cm^−1^ (d), due to P = O stretching, and a peak at 889 cm^−1^ (e), due to P-O bending in chitosan nanoparticles, which is not present in native chitosan, clearly demonstrates the crosslinking of TPP resulting in nanoparticles (34).

The zeta potential of nanoparticles ranged from 25 to 41 mV (Figure 1C). The highest value was determined for unloaded NP (41 mV) and was similar to values measured for NP with lower extract content (~39 mV). The zeta potential value above ± 30 mV indicates greater stability and reduced aggregation, due to the higher repulsion between the particles (45). Therefore, those particles will be suitable for the preparation of stable suspensions. Moreover, the zeta potential was positive for all prepared samples due to positively charged amine groups in chitosan indicating good interaction with the bacterial cell membrane, which usually has a negative charge (38). The particle size and was also confirmed by FE-SEM images (Figure 1D).

The total encapsulation efficiency ranged between 44.65 ± 4.39% to 94.44 ± 0.93%. Only the major cannabinoids in both acidic and decarboxylated forms were detectable in the supernatant (Table 4). The absolute mass of cannabis extract encapsulated in nanoparticles, related to the amount of CS and TPP (3:1 w/w), as well as the extract:chitosan ratio, ranged from 29.55 ± 0.45 mg/g for CNG2 to 56.09 ± 1.88 for CNT1. Nanoparticles with an extract:chitosan ratio of 1:10 (w/w) exhibited a lower EE compared to nanoparticles with a ratio of 1:20 (w/w). On the other hand, the absolute amounts of encapsulated extracts were higher for all encapsulated samples at a 1:10 ratio than for nanoparticles at 1:20 ratio, as is shown in Table 4. Cannabigerolic acid was the only cannabinoid identified in all supernatants simultaneously showing relatively high EE. On the other hand, for the remaining trace cannabinoids, a strong encapsulation rate can also be expected. CBG together with CBDA showed a relatively lower EE compared to CBGA and THC(A). Interestingly the supernatant from CBDA dominant extract (Jonas 1) contained a higher concentration of CBD compared to crude extract. This could be explained by the decarboxylation of CBDA to CBD due to the higher temperature and the lowered pH during preparation of nanoparticles (46).

**Table 4:** Encapsulation efficiency (%) of cannabis extracts.

	Extract:chitosan ratio (w/w)	Cannabinoids						Total EE	Absolute mass ratio of extract:NP (mg/g)
		CBD	CBDA	CBG	CBGA	THC	THCA-A		
CNT1	1:10	-	80.46 ± 5.06^a^	-	72.69 ± 4.72^a^	83.59 ± 0.66^a^	61.48 ± 3.16^a^	74.55 ± 2.51^a^	56.09 ± 1.88^a^
CNT2	1:20	-	-	-	92.44 ± 1.16^b^	96.64 ± 0.23^b^	94.23 ± 1.40^b^	94.44 ± 0.93^b^	35.49 ± 0.38^b^
CNB1	1:10	- 62.45 ± 6.15*	36.49 ± 0.92^b^	-	98.32 ± 0.41^c^	-	-	67.40 ± 0.66^c^	50.68 ± 0.53^c^
CNB2	1:20	- 78.08 ± 3.48*	68.73 ± 0.28^c^	-	99.49 ± 0.20^d^	-	-	84.11 ± 0.24^d^	31.73 ± 0.08^d^
CNG1	1:10	-	13.73 ± 7.14^d^	25.45 ± 5.08^a^	94.78 ± 0.96^e^	-	-	44.65 ± 4.39^e^	34.39 ± 1.65^b^
CNG2	1:20	-	71.43 ± 2.25^e^	66.11 ± 1.31^b^	98.08 ± 0.04^f^	-	-	78.54 ± 1.20^f^	29.55 ± 0.45^f^

Results are presented as mean ± standard deviation. The total EE (%) was calculated as an average of all detected cannabinoids in supernatants. CBD, cannabidiol; CBDA, cannabidiolic acid; CBG, cannabigerol; CBGA, cannabigerolic acid; THC, Δ^9^-tetrahydrocannabinol; THCA-A, tetrahydrocannabinolic acid; NP, nanoparticles; “-“, not detected. To form nanoparticles, TPP was added in ratio 3:1 CS:TPP (w/w) as described in section 2.3. *Cannabidiol was probably formed during nanoparticle preparation by decarboxylation of CBDA. Calculated by equation in 2.4. Different letters in the same column indicate significant differences. *p* < 0.05; *n* = 3.

### 3.3 Antibacterial and antifungal activity of crude and encapsulated extract

The crude extracts exhibited high activity against all tested bacteria (Table 5). The most susceptible bacterium was *S. pyogenes* with MIC_80_ 4 μg/mL for all extracts. All extracts inhibited other bacteria by concentrations ranging from 8–16 μg/mL. The MICs are consistent with previous research (37). However, all ethanol extracts were slightly less effective against *S. aureus* compared to isolated THC, CBD and CBG (MIC 0.5–2 μg/mL) (21, 24). Although cannabinoids represent the extract’s major components, other biologically active substances, especially terpenoids, should not be overlooked. The antimicrobial activity of the identified terpenes in extracts has been proven by many studies (47). *β*-myrcene showed high activity against *S. aureus* and *S. epidermidis* (26), while β-caryophyllene has a proven effect against *S. lugdunensis* and *S. saprophyticus* (48). The unloaded nanoparticles and nanoparticles with encapsulated extracts also showed antimicrobial activity at 32–512 μg/mL concentrations. The bacteria were less susceptible to empty CNP and NP loaded with Hemp G extract (MIC_80_ = 256–512 μg/mL). These results confirm that blank chitosan nanoparticles exhibited antimicrobial activity against the tested bacteria. Moreover, in the case of CNG1 and CNG2, the antimicrobial effect was equal to or even lower than that of the blank CNP. These data demonstrate that the encapsulated extract with a high CBGA content was ineffective against the tested bacteria after encapsulation (with the exception of *S. pyogenes*), and the primary antimicrobial effect can be attributed to the chitosan nanoparticles themselves. These findings are consistent with previous studies (34, 49). The strongest antimicrobial activity was demonstrated by CNT1, where MIC_80_ ranged from 32 to 64 μg/mL. Regarding the EE, it is important to highlight that the extracts after encapsulation represented 2.96 ± 0.05–5.61 ± 0.19% of total mass of NPs. Therefore, the extract encapsulation in chitosan slightly increases its antimicrobial activity. Among the control antibiotics used, both controls showed strong antibacterial effects. AMP had stronger antimicrobial activity (0.0625–2 μg/mL) than CLP (2–8 μg/mL), however, the difference between CLP and cannabis extracts was only a few dilutions and in the case of *S. pyogenes* the extracts were comparable to CLP.

**Table 5:** Antibacterial and antifungal activity of crude extract and chitosan nanoparticles contained encapsulated extracts.

Microorganism		**Strain**	MIC_80_ (μg/mL)											
			CNP	**Chocolope**			**Jonas 1**			**Hemp G**			**Antibiotics**	
				Extract	CNT1	CNT2	Extract	CNB1	CNB2	Extract	CNG1	CNG2	AMP	CLP
**Bacteria**	*Staphylococcus aureus*	ATCC 29213	512	8	64	128	16	128	256	16	256	512	1	8
		ATCC 25923	256	8	32	128	8	64	64	16	512	256	0.0625	8
	*Staphylococcus epidermidis*	CCM 50	256	8	64	256	8	128	256	16	256	512	0.0625	8
		CCM 4418	256	8	64	128	8	128	256	16	256	256	2	4
	*Staphylococcus lugdunensis*	CCM 4069	256	16	64	256	16	128	256	16	256	256	0.25	2
	*Staphylococcus saprophyticus*	CCM 2727	256	8	64	128	16	128	256	16	256	512	0.5	4
	*Streptococcus pyogenes*	CCM 4425	256	4	32	64	4	128	256	4	64	256	2	4

			CNP	Extract	CNT1	CNT2	Extract	CNB1	CNB2	Extract	CNG1	CNG2	CLT	TRB
**Dermatophytes**	*Arthroderma insingulare*	CCF 5417	>1,024	256	512	>1,024	512	1,024	>1,024	512	>1,024	>1,024	0.25	0.5
		CCF 5943	>1,024	512	1,024	1,024	256	>1,024	>1,024	256	>1,024	>1,024	0.25	0.0625
	*Epidermophyton floccosum*	CCM 8339	512	128	256	512	64	256	512	512	512	512	0,25	>16
	*Microsporum gypseum*	CCM 8342	>1,024	128	1,024	>1,024	256	1,024	>1,024	256	1,024	>1,024	0.25	0.0313
	*Nannizzia fulva*	CCF 6025	>1,024	64	256	512	256	256	512	256	>1,024	>1,024	0,25	0.0313
		CCF 5338	>1,024	128	256	512	64	256	512	128	512	1,024	0,5	0.0313
		CCF 5782	>1,024	128	1,024	>1,024	256	512	>1,024	256	1,024	>1,024	0.5	1
	*Nannizzia gypsea*	CCF 5215	>1,024	128	256	512	256	256	512	512	1,024	>1,024	0,25	0.0313
	*Trichophyton interdigitale*	CCM 8337	>1,024	64	512	1,024	64	512	1,024	128	1,024	>1,024	0,125	0.0625
	*Trichophyton rubrum*	CCF 4934	>1,024	256	1,024	>1,024	512	1,024	>1,024	512	1,024	>1,024	0.25	0.0625
		CCF 4879	1,024	128	128	512	256	512	>1,024	256	512	>1,024	0,5	0.0313
	*Trichophyton tonsurans*	CCF 4930	1,024	128	512	1,024	256	1,024	>1,024	256	1,024	>1,024	0.25	0.0625

AMP, ampicillin; CLP, chloraphenicol; CLT, clotrimazole; TB, terbinafine; CNP, empty chitosan nanoparticles; CNT1 and CNT2, Chocolope chitosan nanoparticles; CNB1 and CNB2, Jonas 1 chitosan nanoparticles; CNG1 and CNG2, Hemp G chitosan nanoparticles. The amount of extracts presented in nanoparticles regarding the encapsulation efficiency (Table 4) ranged between 29.55 ± 0.45–56.09 ± 1.88 mg/g.

All strains of dermatophytes were less susceptible to crude or encapsulated extracts compared to bacteria. The MIC_80_ values ranged from 64 to 512 μg/mL, with the modus ranging from 128 to 256 μg/mL. The Chl extract was the most potent, followed by J1 and HG, respectively. The most sensitive dermatophytes were *Nannizzia fulva* CCF 5338 and *Trichophyton interdigitale* CCM 8337, which belong to the most common cause of dermatomycoses together with *T. rubrum* (50). The most resistant strains were *Arthroderma insingulare* (CCF 5417 and 5,943) and *T. rubrum* CCF 4934. So far, very limited evidence of cannabis activity against dermatophytes exists. Turner and Elsohly (1981) reported potent effects of CBC and its analogues against *Trichophyton mentagrophytes* (MIC = 6.25–50 μg/mL) (51). In another study, cannabis extracts obtained from THC and CBD-rich cannabis plants using ethanol as a solvent demonstrated antifungal activity (MIC_50_) against *T. mentagrophytes* ranging from 89.37 to 240 μg/mL (52). The pure CNP showed only low or no activity against dermatophytes in tested concentrations (MIC_80_ was in most cases >1,024 μg/mL), even though CNP bactericidal effect is otherwise quite strong. This low antifungal activity of empty chitosan nanoparticles was previously reported (53, 54). The only exception was *E. floccosum* which was significantly inhibited by all NPs in the range of 256–512 μg/mL, while the widely used antibiotic TB was not effective in tested concentrations. However, these findings confirm the partial antifungal and antibacterial activity of pure chitosan nanoparticles against certain pathogenic bacteria and dermatophytes.

Similarly to antibacterial activity, CNT1 was the most potent against all dermatophytes (MIC_80_ = 256–1,024 μg/mL). The antifungal activity of CNB1 was also confirmed and in the case of *Nannizzia fulva* CCF 6025 the activity of CNB1 (MIC_80_ = 256 μg/mL), compared to the crude extract (MIC_80_ = 256 μg/mL) was the same. Based on the encapsulation efficiency (Table 4) and the amount of encapsulated extract, that ranged between 2.96 ± 0.05 to 5.61 ± 0.19% of NPs, we can confirm that the incorporation of cannabis extracts to chitosan nanoparticles improved the antibacterial and especially antifungal activity by reducing the effective concentration of extracts, compared to the crude form, by more than 20 times in some cases.

### 3.4 Cytotoxicity of crude and encapsulated extracts on human keratinocytes

**Figure 2:**
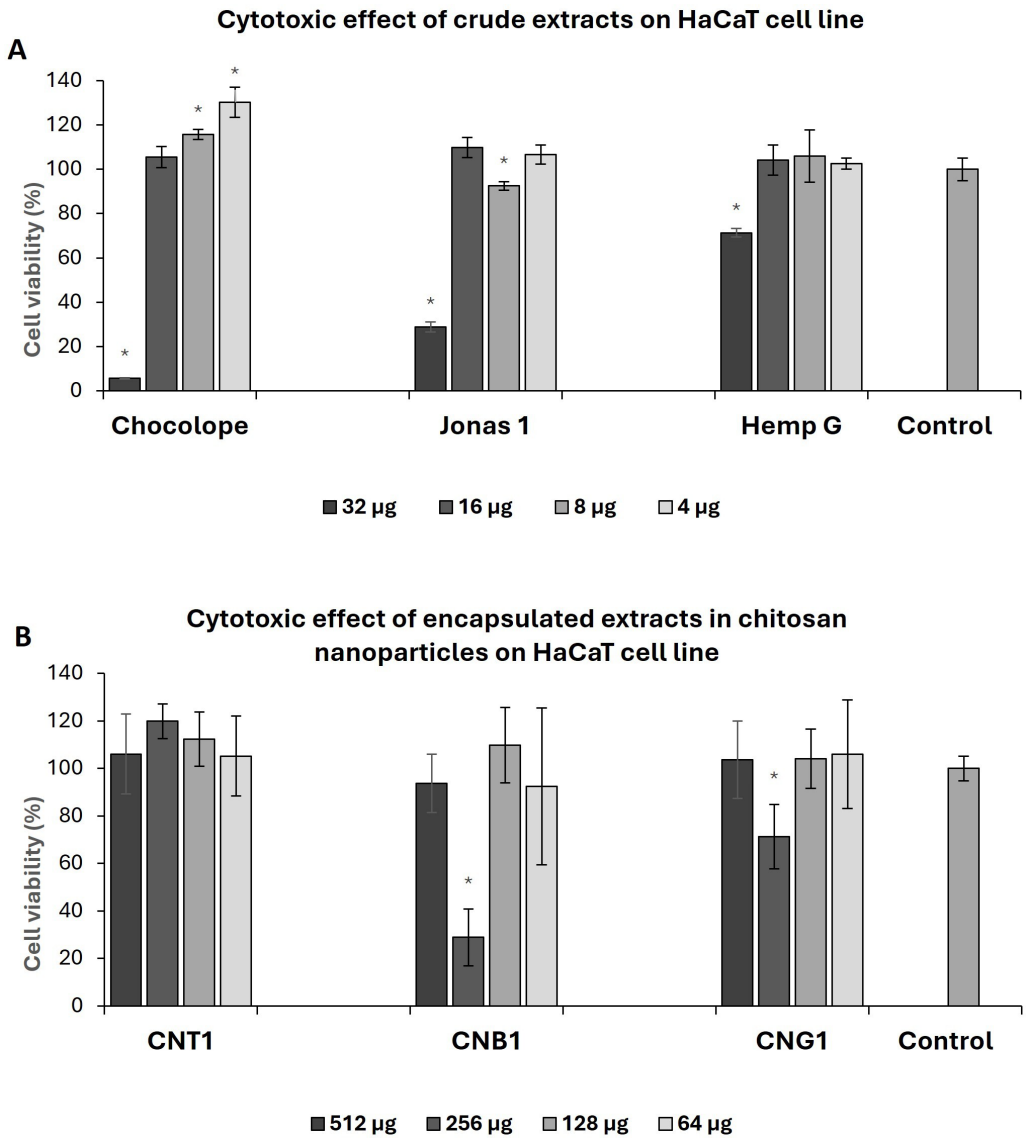
Effect of pure extract on HaCaT cell line after 72 h exposure **(A)**, effect of chitosan nanoparticles with encapsulated extract on HaCaT cell line after 72 h of exposure **(B)**. CNP, empty chitosan nanoparticles; CNT1, Chocolope chitosan nanoparticles; CNB, Jonas 1 chitosan nanoparticles; CNG, Hemp G chitosan nanoparticles. Data are represented as means of control percentage with standard deviations. Values significantly different from the control are marked with asterisks, *p* < 0.05, *n* = 5.

As shown in Table 6, all crude extracts demonstrated moderate cytotoxicity to keratinocytes which increased with time. In general, the extracts were less cytotoxic than isolated compounds, i.e., previously reported IC_50_ values for CBD were 1.83 μg/mL (55), CBG 3.7 μg/mL and CBGA 7 μg/mL (56). In a study involving several cell lines, the cytotoxicity of high-THC ethanolic extracts ranged from 8 to 49 μg/mL after 24 h exposure (57). In a paper investigating the effect of THC on skin protection against UV, concentrations of 1.25 μg/mL were found to be non-cytotoxic. In addition, the authors confirmed slightly increased cell viability (58). This is consistent with our observation as is shown in Figure 2A Pure Chl extract increased viability of keratinocytes by 15.64 ± 6.82% at a concentration of 8 μg/mL compared to control and even by 30.23 ± 3.39% at a concentration of 4 μg/mL, respectively.

**Table 6:** Inhibitory concentration (IC_50_) of cannabis pure and encapsulated extracts in chitosan nanoparticles (μg/mL) on HaCaT cell line.

	IC_50_ (μg/mL)									
	**CNP**	**Chocolope**			**Jonas 1**			**Hemp G**		
		Extract	CNT1	CNT2	Extract	CNB1	CNB2	Extract	CNG1	CNG2
24 h	1713.64 ± 279.06^ab^	60.32 ± 1.24^a^	774.81 ± 73.29^a^	1245.46 ± 135.78^a^	70.30 ± 1.48^a^	653.942 ± 71.98^a^	1059.66 ± 104.98^ab^	72.46 ± 2.55^a^	734.81 ± 73.15^a^	1539.36 ± 122.79^a^
48 h	2190.22 ± 367.19^b^	38.56 ± 0.35^b^	823.86 ± 119.10^a^	1042.26 ± 160.96^a^	61.25 ± 5.30^b^	638.72 ± 68.11^a^	953.30 ± 33.00^a^	69.06 ± 4.14^a^	927.44 ± 132.15^b^	1868.81 ± 78.22^b^
72 h	1593.22 ± 145.86^a^	33.95 ± 1.19^c^	1,253 ± 146.00^b^	1,312 ± 407.80^a^	34.03 ± 0.42^c^	1155.80 ± 267.54^b^	1276.99 ± 186.32^b^	39.58 ± 1.58^b^	1236.24 ± 322.41^c^	2374.92 ± 380.81^c^

CNP, empty chitosan nanoparticles; CNT1 and CNT2, Chocolope chitosan nanoparticles; CNB1 and CNB2, Jonas 1 chitosan nanoparticles; CNG1 and CNG2, Hemp G chitosan nanoparticles. Data are presented as mean ± standard deviation. Different letters in the same column indicate significant differences. *p* < 0.05; *n* = 5. The amount of extracts presented in nanoparticles regarding the encapsulation efficiency (Table 4) ranged between 29.55 ± 0.45–56.09 ± 1.88 mg/g.

Although nanoparticles were formulated with extracts varying in the spectrum of cannabinoids, the overall effect of NPs on keratinocytes was considered as non-cytotoxic as is shown in Table 6. In a paper reported by Ridolfi et al. (2012), a concentration of 500 μg/mL CNP did not affect the viability of keratinocytes after 24 h period, which is in the agreement with our results (59). After exposure of 24 h, the highest cytotoxic effect was observed for CNB1, where IC_50_ was 653.942 ± 71.98 μg/mL, followed by CNG1 (734.81 ± 73.15 μg/mL) and CNT1 (774.81 ± 73.29 μg/mL). Regarding to EE, there was even a slight increase of cytotoxicity effect in some cases compared to pure extracts after 24 h and 48 h exposure period. However, the IC_50_ of nanoparticles decreased with the exposure period as opposed to pure extracts, particularly after 72 h. In addition, regarding EE, the encapsulation reduced the cytotoxic effect of the crude extracts by up to 10 times after 72 h. Moreover, a similar effect on cell viability was observed for the chitosan nanoparticles with high-THC. Enhanced cell vitality by 19.88 ± 7.25% was observed for CNT1 after 72 h of exposure compared to control at a concentration of 256 μg/mL and 12.36 ± 11.4% at a concentration of 128 μg/mL, respectively, contributing to the evidence of a positive effect of THC on skin cells as was described above (Figure 2B). Unlike antibiotics, the effect of cannabis extracts is not limited to antimicrobial activity, since it may have several added benefits. For example, cannabinoids interact with CB1R and CB2R receptors in keratinocytes, which reduce the production of inflammatory factors (TNF-*α*, IL-1, or IL-6) that play an important role in wound healing (60). Furthermore, broad-spectrum extracts may contain other biologically active compounds that can contribute to the advanced effects of encapsulated extracts. These compounds may include terpenes that allow access of lipophilic cannabinoids to deeper layers of the skin or may enhance their activity. This “so called” entourage effect has been demonstrated for some terpenes identified in cannabis (61).

Although crude extracts exhibited moderate cellular cytotoxicity, *in vitro* models cannot completely reproduce human skin and its complex functions. Moreover, topical applications of cannabinoids are now commonly used in medical therapy and have negligible side effects (62). A review by Martins et al. (2022) shows that topical application of cannabinoids is very well tolerated across patients with a range of skin disorders, whereas the treatment period is usually several weeks (63). This again highlights the low cytotoxicity of the extracts *in vivo*.

Chitosan nanoparticles represent a very simple, inexpensive and effective way to apply drugs to the skin, improving their bioavailability by prolonging the dwelling time of topically applied drugs. Additionally, it could enhance the passage of the agent through epithelial cells by opening tight junctions between epithelial cells, while reducing their side effects (64). Moreover, encapsulation of extracts not only improves their application but also reduces their cytotoxic effect on keratinocytes.

## 4 Conclusion

To the best of our knowledge, the present work is the first that demonstrates the successful encapsulation of cannabis extracts into chitosan nanoparticles. This study also provides, for the first time, a characterization of the obtained nanoparticles and determines their *in vitro* antibacterial, antifungal, and cytotoxic activity. Cannabis is known to have a wide range of medical uses, including the treatment of skin diseases; however, limited attention has been paid to its antimicrobial and wound-healing properties. Although crude extracts appeared more effective based on absolute MIC₈₀ values, the actual amount of encapsulated extract needed to inhibit microbial growth was mostly lower, particularly in the case of dermatophytes, where antifungal activity was markedly enhanced. Furthermore, synergic interactions between the extracts and chitosan nanoparticles were observed, leading to stronger antifungal effects than those achieved by the pure extract or empty nanoparticles alone.

Cannabis preparations for topical application are mostly used in the form of gels or ointments. However, the physicochemical properties of the extract itself often pose challenges, limiting its direct application. We have demonstrated here a promising approach to overcome these limitations by encapsulation in chitosan nanoparticles. This method could help to improve the manipulation of the cannabis extracts – i.e., the nano-encapsulated powder is handled better than the original sticky substance. Although encapsulation of the extracts into chitosan nanoparticles primarily enhanced antifungal activity, the antibacterial activity of the prepared nanoparticles increased only slightly. In the case of the high-CBGA extract, no improvement was mostly observed, and the inhibition can be attributed to the chitosan nanoparticles themselves, which possess proven antimicrobial properties. Encapsulation also might enhance the stability and bioavailability of cannabis extracts and leverage the beneficial properties of chitosan, such as its moisturizing and anti-inflammatory effects. Considering that the encapsulated extracts exhibited lower toxicity, as well as the improvement in the metabolic activity of skin cells, the encapsulation of these extracts in chitosan nanoparticles matrices is presented as an suitable alternative therapy for the treatment of skin infections, being able to improve even the wound healing ability and patient comfort.

## Data Availability

The original contributions presented in the study are included in the article/supplementary material, further inquiries can be directed to the corresponding author.
